# The Population Divergence and Genetic Basis of Local Adaptation of Wild Soybean (*Glycine soja*) in China

**DOI:** 10.3390/plants12244128

**Published:** 2023-12-11

**Authors:** Xiaodong Liu, Peiyuan Li, Xiaoyang Ding, Ying Wang, Guangxun Qi, Jiaxin Yu, Yong Zeng, Dezhi Cai, Xuhang Yang, Jiahui Yang, Chunming Xu, Bao Liu, Yingshan Dong, Na Zhao

**Affiliations:** 1 Department of Agronomy, Jilin Agricultural University, Changchun 130118, China; liuxd214@foxmail.com (X.L.);; 2 Jilin Academy of Agricultural Sciences, Changchun 130033, Chinaqiguangxunyl@163.com (G.Q.); yangjiahui940529@163.com (J.Y.); 3 Key Laboratory of Molecular Epigenetics of the Ministry of Education (MOE), Northeast Normal University, Changchun 130024, China; wangy585@nenu.edu.cn (Y.W.); yujx096@nenu.edu.cn (J.Y.); zengy501@nenu.edu.cn (Y.Z.); baoliu@nenu.edu.cn (B.L.)

**Keywords:** wild soybean, *Glycine soja*, adaptation, population divergence

## Abstract

*Glycine soja* is the wild relative species of cultivated soybean. In this study, we investigated the population divergence and genetic basis of the local adaptation of wild soybean in China using genome-wide single-nucleotide polymorphisms (SNPs) of a population of 72 *G. soja* accessions. Using phylogenetic analysis, we observed that *G. soja* accessions clustered into three distinct groups, each corresponding to a specific geographic region, the northeastern region (NER), central region (CR), and southern region (SR), consistent with previous studies. Notably, we found a significant positive correlation between genetic and geographic distances. Further population structure analysis revealed each group was associated with an ancestral population and a specific geographic area. By utilizing the genome sequencing data of accessions from 16 different locations, we inferred the population history of these wild soybean groups. Our results indicate that the three groups diverged ~25,000 years ago, coinciding with the time of the last glacial maximum. The effective population size of the SR group expanded first, and subsequently, the NER and CR groups expanded approximately 5000 and 2500 years ago, respectively. Moreover, 83, 104, and 101 significant associated loci (SALs) were identified using genome-wide association analysis for annual mean temperature, annual precipitation, and latitude, respectively. Functional analysis of genes located in SALs highlighted candidate genes related to local adaptation. This study highlights the significant role of geographic isolation and environmental factors in shaping the genetic structure and adaptability of wild soybean populations. Furthermore, it emphasizes the value of wild soybean as a crucial genetic resource for enhancing the adaptability of cultivated soybeans, which have experienced a loss of genetic diversity due to domestication and intensive breeding practices. The insights gained from our research provide valuable information for the protection, conservation, and utilization of this important genetic resource.

## 1. Introduction

Crop wild relatives (CWRs) are widely recognized as invaluable genetic resources for crop enhancement [1,2,3]. Among these, wild soybean (*Glycine soja*) is a notable relative of the cultivated soybean. Predominantly found in East Asia, wild soybean spans regions including China, Korea, Japan, and parts of Russia [4]. China, in particular, boasts a wide distribution of wild soybeans and has accumulated a rich genetic diversity [5].

Early studies have demonstrated that the distribution of wild soybeans within China can be primarily categorized into three ecological regions: northeastern China, the Huang-Huai Valley, and southern China [6,7]. One study indicated that the southern group possesses the highest genetic diversity of *G. soja*, while the central group exhibits the lowest among the three Chinese *G. soja* groups [8]. Significant genetic differentiation has been observed among these groups from the three ecological regions [8]. The genomic diversity of *G. soja* demonstrates a distinct geographical pattern, shaped by the interplay of natural selection, gene flow, and genetic drift [8]. Further research has unveiled a significant positive correlation between geographic distance and genetic distance among *G. soja* accessions, underscoring the impact of isolation by distance on genetic diversity [9]. Leamy et al. [10] proposed that *G. soja* survived in multiple cryptic refugia in China during the last glacial maximum (2.2 × 10^4^ years ago) and began to expand and diverged due to the heterogeneity of the environment. He et al. [11] proposed that the main refugia of *G. soja* were mainly in southern China during the LGM and then *G. soja* gradually expanded from the southern to the northern region. An earlier study on the population structure of the wild soybean in China demonstrated that the northeastern China group and southern China group show a similar ancestral genotypic origin, while the Huang-Huai group is independent [6]. Guo et al. [6] proposed that there was an expansion event of *G. soja* across the East China Sea land bridge during the LGM, and the insufficient lineage sorting and differentiation after expansion resulted in similar genotypes in northeastern and southern China despite their geographical separation. Recently, demographic analysis of the *G. soja* population revealed a divergence amongst wild soybeans from these three ecological zones approximately 100,000 years ago, followed by varying degrees of population expansion, which support the expansion in the LGM [9]. Previous studies did not detect significant bottlenecks in *G. soja* during the LGP [9,12]. More evidence is still required to establish how the *G. soja* population expanded and diverged in China.

Natural selection has been instrumental in shaping the adaptation of *G. soja* varieties tolerant to specific environmental stresses such as drought, salinity, pests, and disease [11,13,14,15,16]. The species’ broad geographic distribution and resilience to harsh environmental conditions suggest a wealth of adaptive genes within *G. soja* [9,11]. Recent research has pinpointed several genes involved in local adaptation, such as those related to flowering time and temperature, using genome–environment associations [9]. A recent study suggests that the introgression from adapted sympatric wild soybeans facilitated the local adaptation of landraces during the expansion of cultivated soybean [17]. Numerous beneficial genes or genetic loci have been identified from *G. soja* resources, including those conferring abiotic resistance [18,19,20,21], biotic resistance [22], and adaptation to various environmental conditions [23,24,25,26]. Consequently, wild soybean represents a valuable gene reservoir for the genetic enhancement of cultivated soybeans, providing them with the capacity to adapt to new environments.

Wild soybean is primarily found in open habitats with frequent human activity. Unfortunately, the distribution of wild soybean has significantly declined, with numerous populations becoming extinct or significantly reduced, which is largely due to the fragmentation and reduction in their natural habitats caused by land exploitation and utilization in many locations [11]. Moreover, the expansion of cultivated soybean has further compressed the natural *G. soja* genetic resources. A recent study reported the natural hybridization between transgenic and wild soybean genotypes, presenting new challenges for the protection of *G. soja* [27]. Consequently, it is of vital importance to conserve the genetic resources of wild soybean. Recent studies have shown that genomic introgression between *G. soja* and *G. max* occurred repeatedly during soybean domestication and expansion [17,28]. Mixing the genome of cultivated soybean may further complicate the study of wild soybean evolutionary history.

In this study, we conducted a comprehensive genomic investigation on 72 *G. soja* accessions collected from 16 conservation regions that are isolated from human agricultural activities as part of a wild soybean ex situ protection program. We analyzed the phylogenetic relationships, population structure, selective sweeps, and demographic history of the wild soybean population. Importantly, we identified several genetic loci and explored the candidate genes that were significantly associated with environmental factors using association analysis. Our findings may facilitate the conservation of wild soybean genetic resources and provide fundamental insights for the further improvement of cultivated soybean to adapt to the rapidly changing global climate.

## 2. Results

### 2.1. Phenotypic Variation in the Wild Soybean Population

The 72 *G. soja* accessions collected from the northeastern region (NER), Huang-Huai Valley (central region (CR)), and southern region (SR) of China were grown in pots in 2016 (Supplementary Table S1; Figure S2). Eleven traits of 64 accessions were recorded. We found all wild soybean lines displayed a purple flower color, brown pubescence color, and black seed coat color. Most lines exhibited a bloom seed coat (95%), no seed luster (91%), a flat ellipse seed shape (84%), and a black hilum color (81%) (Table 1). Leaf shape, the type of pubescence, and pod color showed high polymorphism in the wild soybean population. The number of accessions with lanceolate leaves was the lowest (11 accessions), while that with ellipse-shaped leaves was the highest (37 accessions), and there were 22 accessions with ovoid-shaped leaves (Table 1). For the pubescence type, 37 accessions showed oblique pubescence, 22 accessions had flat pubescence, and 5 accessions had erect pubescence (Table 1). Regarding pod color, 30 accessions exhibited dark brown pods, while 18 and 16 accessions showed brown and black pods, respectively (Table 1). Compared with the other two groups, the SR exhibited higher diversity in the traits of pubescence type and seed coat bloom (Table 1).

### 2.2. Genetic Divergence and Geographic Population Structure in Wild Soybean

**Figure 1:**
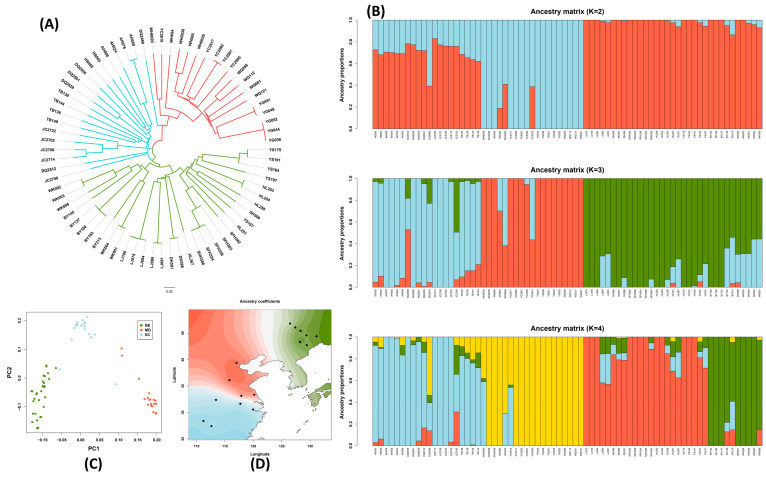
Phylogenetic tree, principal component analysis (PCA), population structure, and spatial interpolation of ancestry coefficients in wild soybean. (**A**) The phylogenetic tree of wild soybean samples derived using the neighbor-joining method. Branch colors represent different eco-regions: green is northeast region (NER), red is central region (CR), and light blue is southern region (SR). (**B**) The population structure of wild soybean for K = 2, K = 3, and K = 4. The accessions are ordered (left to right) by latitudes of collecting locations from low to high. Different colors represents different ancestry coefficients. (**C**) PCA plot for wild soybean samples. Each dot represents one sample, and different eco-regions are shown in different colors. (**D**) Geographic maps of ancestry coefficients for K = 3 ancestral populations. Dots indicate the geographic origins of samples. Different eco-regions are shown in different colors. The higher the color shade higher the percentage of membership.

We analyzed the phylogenetic relationships of the wild soybean population using genome-wide SNPs. The phylogenetic tree demonstrated that the accessions can be divided into three groups, which align closely with the geographical distribution (Figure 1A). Most samples from the NER, CR, and SR are clustered into different groups (Figure 1A). We calculated the genetic distances between the 16 locations and performed a Mantel test to examine the correlation between the genetic distance matrix and geographical distance matrix (based on the GPS coordinates) (Supplementary Table S2). A significant positive correlation was found (with a Mantel test *p*-value of <0.001), suggesting that the genetic diversity follows the geographical pattern of distribution.

The wild soybean population structure was explored using ADMIXTURE (version 1.3) and the best K (K = 3) was determined based on the error rates in five-fold cross-validations. When K = 3, the models fit most samples from the three eco-regions as each being derived from three distinct ancestral populations, which aligns with the phylogenetic results. The three distinct groups largely correspond to the three geographic regions (Figure 1B). We further explored the ancestral coefficients for K = 2 and K = 3 for wild soybean accessions. When K = 2, the NER accessions and CR accessions were estimated to be derived from two different source populations, while the SR accessions shared the majority component of the ancestral population with the NER accessions, even though they were admixed with the CR pool (Figure 1B). Further analysis based on K = 4 showed that the NER accessions were estimated to be derived from two different source populations, while the CR accessions and SR accessions showed a similar ancestral admixture as K = 3 (Figure 1B). Consistently, the principal component analysis (PCA) also showed that the wild soybean accessions from the three eco-regions formed three groups correspondingly (Figure 1C). Spatial interpolation analysis was performed to visualize the ancestry coefficients (Q matrix) on a geographic map, further addressing the geographic distribution of the population structure (Figure 1D). The interpolated ancestry for K = 3 presented strong geospatial overlap with the three known ecoregions: northeastern, central, and southern China (Figure 1C). This suggests a potential role of geographical isolation in shaping the genetic structure of wild soybean populations.

### 2.3. Selection Signatures between Genetic Groups and the Genetic Loci Associated with Local Adaptation in G. soja

**Figure 2:**
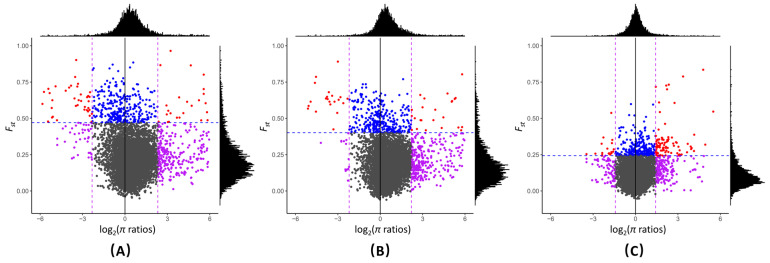
Distribution of log-transferred θπ ratios and F_ST_ values, which are calculated in 500 kb windows sliding in 100 kb steps between groups. (**A**): CR and NER, (**B**): CR and SR, and (**C**): NER and SR. Points (purple color) located to the left and right vertical dashed lines correspond to the 5% left and right tails of the log-transferred *π* ratio distribution, and points (blue color) above the horizontal dashed line correspond to the 5% right tail of the empirical *F_ST_* distribution. The overlapping regions of both 5% of log-transferred θπ ratio and *F_ST_* were identified as selected regions between groups (red color). NER, northeastern region; CR, central region; and SR, southern region.

We used a method that combined the high-fixation index *F_ST_* (top or low 5%) and high difference in genetic diversity (*π* ratio 5%) to identify selective sweep regions between different *G. soja* groups. We identified 29, 24, and 48 selective sweeps in CR vs. NER, CR vs. SR, and NER vs. SR, respectively (Figure 2, Supplementary Table S3). Gene Ontology (GO) enrichment analysis of genes within the selective sweeps revealed that ATPase activity, coupled with the transmembrane movement of substances (*p*-value = 1.17 ×10^−6^; q-value = 0.001), was significantly over-represented in genes in selective sweeps in NER vs. SR. No over-represented GO terms were identified in genes in the selective sweeps in CR vs. NER and CR vs. SR.

**Figure 3:**
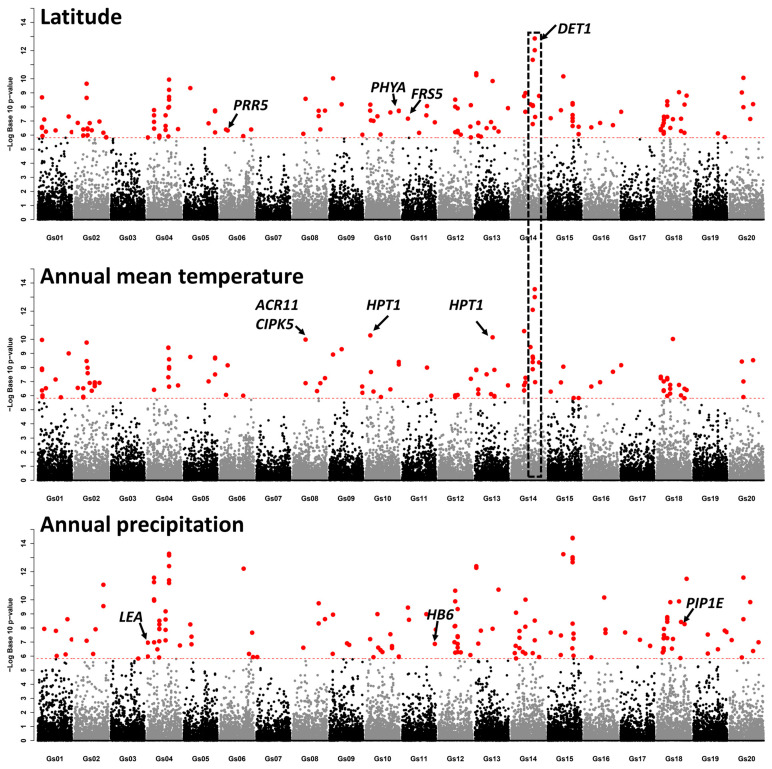
Manhattan plot of genome-wide association analysis. X-axis is position on each chromosome. Y-axis is the log10-transferred *p*-values. Each dot represents an SNP. Red dashed line indicates the cutoff (q-value < 0.05). The significant SNPs are shown in red. *PSEUDO-RESPONSE REGULATOR 5* (*PRR5*), *PHYTOCHROME A* (*PHYA*), *FAR1-RELATED SEQUENCE 5* (*FRS5*), *DE-ETIOLATED1* (*DET1*), *ACT DOMAIN REPEATS 11* (*ACR11*), *CBL-INTERACTING PROTEIN KINASE 5* (*CIPK5*), *HOMOGENTISATE PHYTYLTRANSFERASE 1* (*HPT1*), *DEHYDRIN LEA* (*LEA*), *HOMEOBOX PROTEIN 6* (*HB6*), and *PLASMA MEMBRANE INTRINSIC PROTEIN 1E* (*PIPE*).

To further explore the genes involved in local adaption, we performed a genome-wide association analysis for three ecological factors: latitude, annual mean temperature, and precipitation. We identified 82, 104, and 101 significantly associated loci (SAL) for annual mean temperature, annual precipitation, and latitude, respectively (Supplementary Dataset S1–S3). We further analyzed the functions of *Arabidopsis* homologs for genes located in SALs (Supplementary Dataset S4–S6. Two homologs of *Arabidopsis thaliana HOMOGENTISATE PHYTYLTRANSFERASE 1* (*HPT1*) were found in a SAL on chromosome 13 (Gs13:26226016–26434463, 1.48 ×10^−8^) and a SAL on chromosome 10 (Gs10:7082290–7282290, 5.26 ×10^−11^), respectively (Figure 3). *HPT1* is known to play a role in the adaptation to low-temperature stress [29]. Additionally, homologs of *A. thaliana ACT DOMAIN REPEATS 11* (*ACR11*) and *CBL-INTERACTING PROTEIN KINASE 5* (*CIPK5*) were found in the SAL on chromosome 8 (Gs08:17994333–18194356; *p*-value = 1.04 ×10^−10^) (Figure 3). *ACR11* has been reported to be involved in the response to cold [30], and *CIPKs* play an important role in the signaling module of different stresses [31,32]. Overexpressed *CIPKs* resulted in better tolerance to different stresses compared with wild-type plants in several important crops [33,34,35]. A homolog of *A. thaliana DE-ETIOLATED1* (*DET1*) was found in the most significant SAL for latitude (Gs14:34726425–34926427; *p*-value = 1.37 ×10^−13^) (Figure 3). *DET1* suppresses flowering in short-day conditions and thus plays an important role in maintaining photoperiod sensitivity in *Arabidopsis*. Several other genes, including *PRR5*, *PHYA*, and *FRS5*, which are implicated in the regulation of flowering or photomorphogenesis, were also detected among the SALs related to latitude (Figure 3). Homologs of *A. thaliana PLASMA MEMBRANE INTRINSIC PROTEIN 1E* (*PIPE*), *DEHYDRIN LEA* (*LEA*), and *HOMEOBOX PROTEIN 6* (*HB6*) were found in SALs for annual precipitation (Figure 3). These genes are known to play roles in the response to water deprivation or the water response. These findings provide valuable insights into the genetic basis of local adaptation in wild soybean populations and could be beneficial for future soybean breeding efforts.

### 2.4. The Demographic History of the Three G. soja Genetic Groups

**Figure 4:**
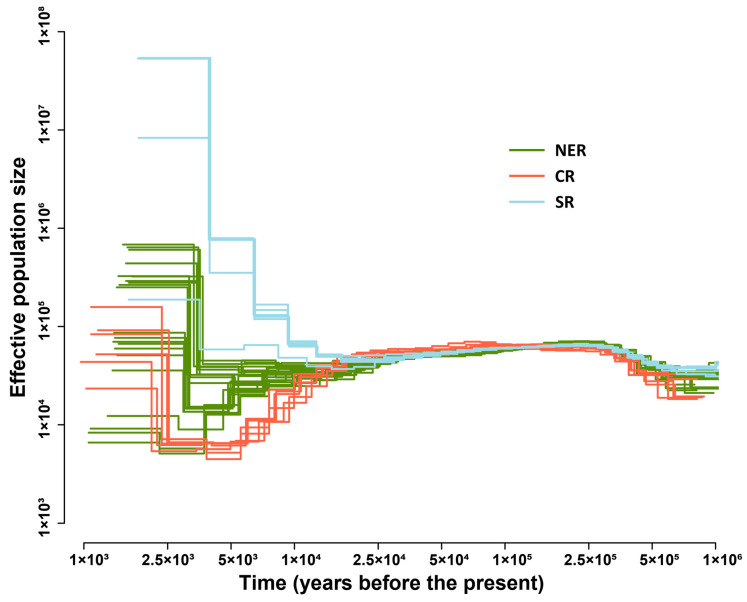
Effective population size history of *G. soja* assessed using MSMC2. The X-axis is the time before the present. The Y-axis is the estimated effective population size.

We performed whole-genome resequencing of one sample from each of the 16 habitats to explore the demographic history of the three *G. soja* groups. We evaluated the expansion time of the effective population size of each *G. soja* group using the MSMC2 based on whole-genome sequencing (WGS) data (Figure 4). The inferred demographic history suggests that the three groups diverged approximately 25,000 years ago. The effective population size (*Ne*) of the SR group expanded first. Subsequently, the *Ne* of the NER group expanded approximately 5000 years ago (Figure 4). The CR group experienced a decrease in effective population size and expanded approximately 2500 years ago (Figure 4). The effective population expansions were to different degrees in the three *G. soja* groups. The SR group expanded to a higher degree than in the other groups. This demographic history provides valuable insights into the population dynamics of the three *G. soja* groups.

## 3. Discussion

The expansion and divergence history of *G. soja* holds significant implications for wild soybean evolution and conservation. Early studies using microsatellites have revealed three geographically distinct *G. soja* genetic groups [36,37]. Subsequent studies have further elucidated the population structure of *G. soja* accessions in China, revealing that accessions from northeastern China and southern China share a similar ancestral genotypic origin, while those from the Huang-Huai valley are independent [6]. Another study identified four genetic groups that largely corresponded to the geographic regions of central China, northern China, Korea, and Japan, with high levels of admixture between genetic groups [10]. In a recent study, the northeastern accessions were further clustered into two groups divided by latitude in 48° N [9]. In this study, when two ancestral populations (K = 2) were assumed, the NER and CR accessions showed different ancestral genetic origins, while SR showed an admixture of two ancestral components, and the major ancestral component of SR was the same as NER’s (Figure 1B), which is in line with the findings of Guo et al. 2012 [6]. When K = 4, we found the NER group was divided into two groups along the latitude, which is consistent with the findings of a recent study by Wang et al. in 2022 [9]. The geographic patterns of the population structure have been explained by the population expansion and divergence during the LGM [6,9,10,11]. The recent study based on the whole-genome resequencing data of 185 diverse wild soybean accessions collected from three major agroecological zones in China showed that *G. soja* groups diverged ~100,000 years ago, and then the population size of each group expanded to different degrees, which supports the hypothesis of expansion in the LGM [9]. However, no study detected significant bottlenecks in *G. soja* during the LGM [9,12]. Herein, we found the divergence of the three *G. soja* groups to be ~2.5 × 10^4^ years ago, which is more consistent with the time of the LGM [38]. Furthermore, we found the effective population size of the three *G. soja* groups expanded to different times and degrees. The effective population size of the SR group expanded first and highest among the three groups, and then the effective population size of NER expanded ~5 × 10^3^ years ago. Interestingly, we observed that the CR group experienced an obvious decline in effective population size and then expanded ~2.5 × 10^3^ years ago. Our study provides more evidence for uncovering the history of the expansion and divergence of *G. soja* as well as the implications for the protection and conservation of this important genetic resource.

Soybean is an important crop that is a leading source of dietary protein and oil in the world. Cultivated soybean was domesticated from annual wild soybean in East Asia 6000–9000 years ago [39]. During its domestication, soybean experienced a genetic “bottle neck”, which resulted in a dramatic loss of genetic diversity [40]. Wild soybean has higher genetic diversity than cultivated soybean [41,42]. Cultivated soybean has lost ~ 50% of its sequence diversity compared with wild soybean [40]. Such low genetic diversity of the domesticated germplasm not only hinders current soybean breeding and improvement efforts but also makes this important crop vulnerable to emerging biotic and abiotic stressors, thus threatening long-term food security [2]. The genetic diversity of CWRs could also be used to decrease the rate of genetic diversity loss, which has been happening over decades of crop domestication and intense breeding [3]. There has been a steady increase in the rate of release of cultivars containing genes from CWRs during the last few decades [1]. Although *G. soja* and *G. max* are primarily selfing plants, there are no reproductive barriers between them; therefore, the genes for adapting to certain environmental conditions can be introduced into the cultivated soybean via artificial hybridization. Recent studies have indicated that soybean landraces migrated to the southern and northern regions of China, and the gene flow from local wild populations possibly accelerated local adaptation [17]. The broad geographic distribution and resilience to harsh environmental conditions suggest a wealth of adaptive genes within *G. soja* [9,11]. Numerous beneficial genes or genetic loci have been identified in *G. soja* resources, including those conferring abiotic resistance [18,19,20,21], biotic resistance [22], and adaptation to various environmental conditions [9,23,24,25,26].

In this study, we explored the genetic basis for the adaptation to local environments by analyzing the association between SNPs and environmental factors. We identified a few SALs for annual mean temperature, annual precipitation, and latitude. A few genes whose *Arabidopsis* homologs have been known to evolve molecular functions or biological processes related to adaptation were explored (Figure 3). Our results indicate that the wild soybean represents a valuable gene reservoir for the genetic enhancement of cultivated soybeans, providing them with the capacity to adapt to new environments.

## 4. Materials and Methods

### 4.1. Plant Materials

The 72 *G. soja* accessions were collected from 16 conservation areas across China between 2006 and 2013 (Table S1; Figure S2). In each location, seeds for accessions that were more than 1 km apart were collected. The seeds were preserved in vacuum packaging and stored at a temperature of −20 °C within the facilities of the Jilin Academy of Agricultural Sciences, Jilin, China. The wild soybean accessions were cultivated in soil-filled pots at the experimental station of the Jilin Academy of Agricultural Sciences. During the V3 stage of growth, the second fresh trifoliate leaf from each accession was harvested and immediately frozen in liquid nitrogen for future analysis.

### 4.2. DNA Exaction, Genotyping by Sequencing (GBS), and Whole-Genome Sequencing (WGS)

DNA was extracted utilizing the cetyltrimethylammonium bromide (CTAB) method. Initially, 1 mL of CTAB buffer was added to 100 mg of ground leaf material, which was then subjected to a 65 °C water bath for a duration of 60 min. Following this, 1 mL of a chloroform/isoamyl alcohol mixture (24:1) was introduced and thoroughly mixed. The resulting mixture was centrifuged at a speed of 12,000 revolutions per minute (rpm) for 10 min, after which the aqueous upper phase was carefully transferred to a fresh microcentrifuge tube. Approximately 1/10 of the aqueous upper-phase volume of 3M sodium acetate, along with 500 µL of cold isopropanol, was added to the tube and incubated at −20 °C for 20 min to precipitate the DNA. The DNA pellet was then collected via centrifugation at 12,000 rpm for 10 min, followed by a wash with 500 µL of ice-cold 70% ethanol. The DNA pellet was finally resuspended in 50 µL of Tris-EDTA buffer. The DNA purification process was completed by repeating the procedure starting from the addition of the chloroform/isoamyl alcohol mixture (24:1). The GBS library construction and sequencing were performed at BGI Genomics Company Limited, Shenzhen, China. The GBS libraries were sequenced on the Illumina HiSeq2000 platform (Illumina, San Diego, CA, USA) with a 100 bp paired-end strategy. After demultiplexing and removing low-quality reads, the average yield of data was ~0.537 gigabase (Gb) for each accession. For each of the 16 locations, one accession was selected for whole-genome sequencing (WGS). The WGS libraries were sequenced on the Illumina HiSeqX platform (Illumina, San Diego, CA, USA) with a 150 bp paired-end strategy. For each accession, ~10 Gb of clean data was sequenced.

### 4.3. Data Processing and Variant Calling

The reads were filtered using Trimmomatic (version 0.39) with a set of parameters “ILLUMINACLIPTruSeq3-PE-2.fa:2:30:10 LEADING:3 TRAILING:3 HEADCROP:5 MINLEN:50”, and only the paired reads that passed the filtration were used for further steps [43]. The reads were then mapped to the reference sequences, which were composed of the *G. soja* genome (v1.0) as well as the mitochondria (NC_039768.1) and chloroplast (NC_022868.1) genomes using BWA (Version: 0.7.17-r1188) [44]. The BAM files were sorted using picard (version 2.25.4, http://broadinstitute.github.io/picard/, accessed on 30 October 2023). Variants were detected and genotyped using BCFtools (version 1.15.1) [45] with the setting “-E -q 10 -Q 20”. The bi-allelic SNPs whose minimum genotyping quality was more than 20 and whose per sample depth was no less than 3 were selected using VCFtools (version 0.1.16) [46]. Furthermore, a max. missing rate of less than 0.5 and minor allele frequency (MAF) of no less than 0.05 were applied to filter the variants. Finally, 48,647 SNPs were left for downstream analysis.

### 4.4. Phylogenetic Tree, PCA, and Population Structure

SNPs were filtered to remove sites whose genotyping rate was less than 90% and then were thinned for an LD of less than 0.1 using PLINK (version 1.90b6.9) [47] with the parameter “--indep-pairwise 50 10 0.1”. Finally, 2633 SNPs were retained for further population genetic analysis. The variants were transformed into fasta sequences for all samples using “vcf2phylip.py” (https://github.com/edgardomortiz/vcf2phylip, accessed on 30 October 2023). The sequences were aligned, and the phylogenetic tree was constructed using the neighbor-joining method in MEGA-X (version 10.2.5) [48]. For the PCA, the eigenvector and eigenvalues were calculated using PLINK (v1.90b6.9) [47]. For the population structure analysis, the ancestry matrices were calculated using ADMIXTURE (version 1.3.0) with 1000 bootstraps [49]. The best k was selected based on the error rates of 5-fold cross-validations from K = 2 to K = 10. The spatial interpolation of ancestry proportions was inferred and displayed using the R package “tess3r” [50].

### 4.5. Mantel Test for Correlation between Genetic Distance and Geographic Distance

The mean genetic distances between the 16 locations were calculated using MEGA-X (version 10.2.5) [48]. The geographic distances based on GPS coordinates were calculated using the R package “geosphere”. The Mantel test to examine the correlation between the genetic distance matrix and the geographical distance matrix was performed using the “mantel.test” function in the R package “ape”.

### 4.6. Identification of Selective Sweeps

The redundant accessions whose genetic distances were < 0.01 were randomly removed to retain only one sample. Finally, 21, 15, and 19 accessions from the NER, CR, and SR groups were retained, respectively. The fixation index (*F_ST_*) and genetic diversity (π) values were calculated for 500 kb windows with a step of 100 kb along each chromosome using VCFtools (version 0.1.16) [46]. The *π* ratios were calculated by comparing the π-values between *G. soja* genetic groups. The windows located in both the 5% left or right tails of the log-transferred π ratio distribution and the 5% right tail of the empirical *F_ST_* distribution were identified as under-selection. The overlapped windows were then merged as the selective sweep regions.

### 4.7. Ecological Association Tests and Significantly Associated Loci (SALs)

The standard climate data were downloaded from WorldClim (https://worldclim.org/data/worldclim21.html, accessed on 30 October 2023). The annual mean temperature and precipitation were averaged for the values in the area of longitude (±0.1) and latitude (±0.1) for each location. The genome-wide association analysis between SNPs and ecological factors was performed based on the latent factor mixed model using the lfmm function in the R package “LEA” [51]. The raw *p*-values were adjusted with the FDR method. SNPs with adjusted *p*-values of less than 0.05 were classified as significantly associated SNPs. Then, the up- and downstream 100 kb regions of the significantly associated SNPs were classified as the associated regions. Furthermore, the overlapping regions were merged as the significantly associated loci (SALs). Genes located in SALs were selected, and homologous genes in *Arabidopsis thaliana* (TAIR10) based on the best hit of NCBI blastp were extracted from the annotation file, which was downloaded from Phytozome.

### 4.8. Inference of Demographic History

The demographic history of the *G. soja* groups was inferred using MSMC2 based on the WGS data. Because *G. soja* is a predominantly selfing species, we adopted a strategy of creating pseudodiploid genomes from data for two accessions, as previously suggested in [9,12,52]. Briefly, the WGS data were mapped onto the reference genome using BWA (Version: 0.7.17-r1188) [44]. Then, the BAM files were used to call genotypes using the “mpileup” function in SAMtools (release 1.2) [53] and the “call” function in BCFtools (release 1.2) [45] with a minimum mapping quality of 20 and a minimum base quality score of 20. “bamCaller.py” and “generate_multihetsep.py” from MSMC-Tools (https://github.com/stschiff/msmc-tools, accessed on 30 October 2023) were used to prepare the mask files and input files for MSMC2. Genotype calls overlapping with repetitive regions in the reference genome were negatively masked. For each accession, one allele at heterozygous sites was randomly chosen. We created pseudodiploid genomes for all possible pairwise combinations of accessions in each group. The MSMC2 was used to infer changes in effective population size (*Ne*) in the *G. soja* groups. The analysis employed default parameters for the MSMC2 program. The effective population size and time were scaled by assuming a mutation rate of 1.5 × 10^−8^ mutations per nucleotide per year [54] and a generation time of 1 year.

## Figures and Tables

**Table 1:** Summary of phenotypes of accessions in different groups.

Trait	Phenotype	NER ^a^	CR ^b^	SR ^c^
Flower color	Purple	32	16	16
Pubescence color	Brown	32	16	16
Seed coat color	Black	32	16	16
Leaf shape	Lanceolate	6	2	2
	Ovoid	15	2	3
	Ellipse	11	12	11
Pubescence type	Erect	0	0	5
	Oblique	15	14	8
	Flat	17	2	3
Seed shape	Round	1	0	0
	Oblate	6	0	1
	Ellipse	1	0	0
	Flat ellipse	24	16	13
	Long ellipse	0	0	2
Hilum color	Yellow	1	1	2
	Light black	1	3	4
	Black	30	12	10
Seed coat bloom	No	0	0	3
	Yes	32	16	13
Seed luster	No	28	11	9
	Weak	4	5	7
Pod shape	Straight	10	3	2
	Bent	22	13	14
Pod color	Brown	5	6	7
	Dark brown	15	7	8
	Black	12	3	1

^a^ NER, northeast region. ^b^ CR, central region. ^c^ SR, southern region.

## Data Availability

The GBS data and WGS data for this study have been submitted to the NCBI SRA database and can be found under the following accession numbers: PRJNA1036371 and PRJNA1036819.

## Supplementary Materials

The following supporting information can be downloaded at https://www.mdpi.com/article/10.3390/plants12244128/s1. Figure S1: Cross-validation plot for different K-values; Table S1: Information of 72 *G. soja* accessions; Figure S2: GPS coordinates of the 16 sampling locations; Table S2: Mean genetic distance between locations; Table S3: Selective sweeps between different *G. soja* groups; Supplementary Dataset S1: SALs for annual mean temperature; Supplementary Dataset S2: SALs for annual mean precipitation; Supplementary Dataset S3: SALs for latitude; Supplementary Dataset S4: The best blastp hits in *Arabidopsis thaliana* for genes in SALs for annual mean temperature; Supplementary Dataset S5: The best blastp hits in *Arabidopsis thaliana* for genes in SALs for annual mean precipitation; Supplementary Dataset S6: The best blastp hits in *Arabidopsis thaliana* for genes in SALs for latitude.
