# Supplementary material for: The Population Divergence and Genetic Basis of Local Adaptation of Wild Soybean (Glycine soja) in China
Source: Plants (Basel). 2023 Dec 11;12(24):4128. doi: 10.3390/plants12244128 (PMC10747053; doi:10.3390/plants12244128)

Figure S1. Cross-validation plot for different K values.

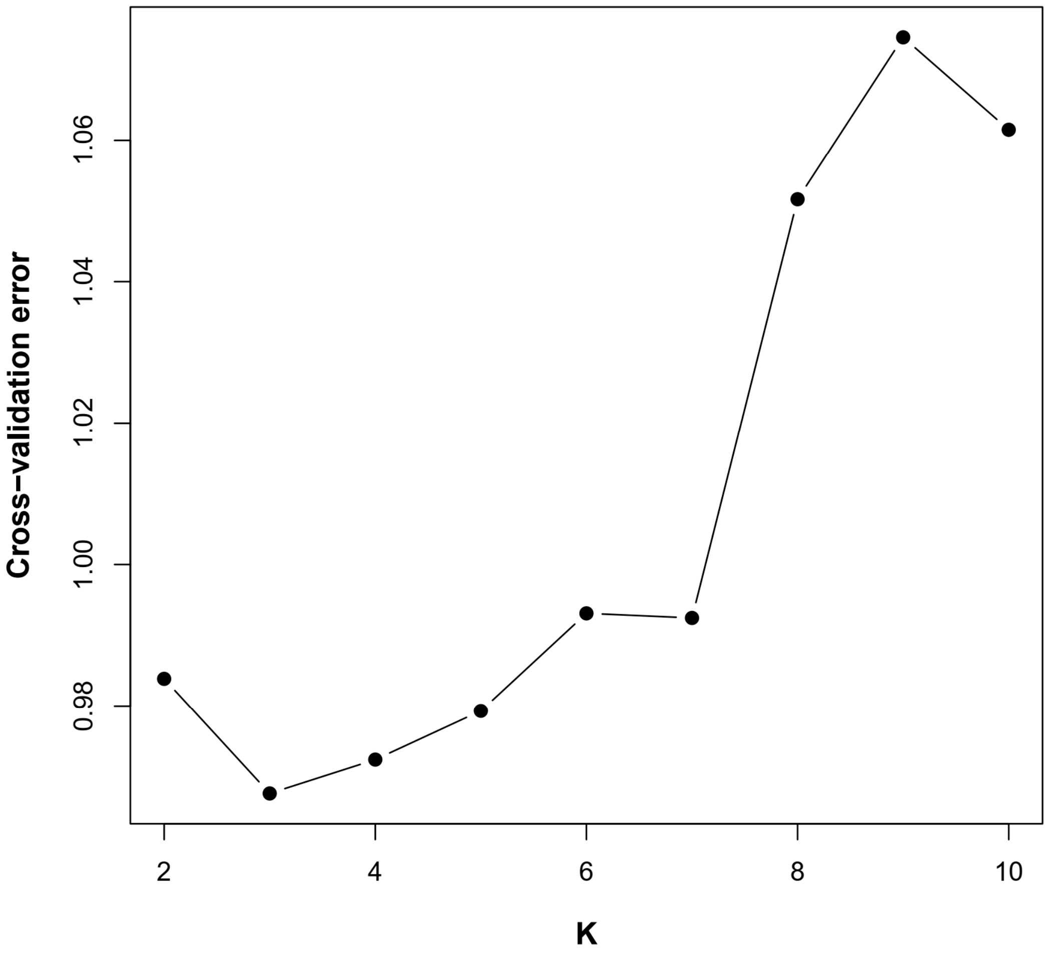

Figure S2. GPS coordinates of the 16 sampling locations.

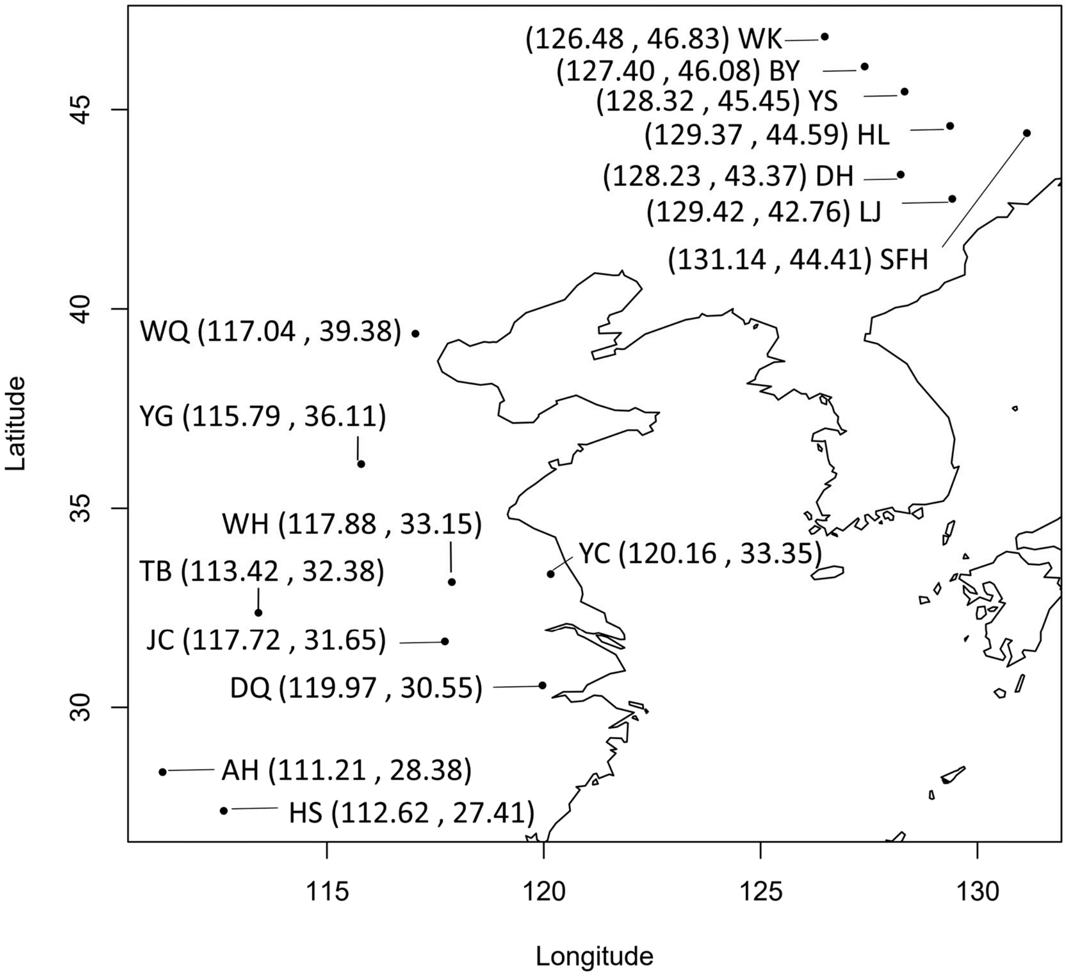

Supplement: Supplementary file 1 [file plants-12-04128-s001.zip › Supplementary Figures.pdf]
