# Supplementary material for: The Population Divergence and Genetic Basis of Local Adaptation of Wild Soybean (Glycine soja) in China
Source: Plants (Basel). 2023 Dec 11;12(24):4128. doi: 10.3390/plants12244128 (PMC10747053; doi:10.3390/plants12244128)
Supplement: Supplementary file 1 [file plants-12-04128-s001.zip › Supplementary Tables.pdf]

Table S1. Information of 72 *G. soja* accessions.

Location ID	Library ID	accession	Eco-region	Location	Latitude	Longitude
YS	301	YS175	NER	Yanshou, Heilongjiang	45.45	128.32
YS	302	YS164	NER	Yanshou, Heilongjiang	45.45	128.32
YS	303	YS157	NER	Yanshou, Heilongjiang	45.45	128.32
YS	304	YS191	NER	Yanshou, Heilongjiang	45.45	128.32
YS	305	YS197	NER	Yanshou, Heilongjiang	45.45	128.32
HL	306	HL267	NER	Hailin, Heilongjiang	44.59	129.37
HL	307	HL258	NER	Hailin, Heilongjiang	44.59	129.37
HL	308	HL253	NER	Hailin, Heilongjiang	44.59	129.37
HL	309	HL251	NER	Hailin, Heilongjiang	44.59	129.37
HL	310	HL254	NER	Hailin, Heilongjiang	44.59	129.37
BY	311	BY193	NER	Bayan, Heilongjiang	46.08	127.4
BY	312	BY180	NER	Bayan, Heilongjiang	46.08	127.4
BY	313	BY213	NER	Bayan, Heilongjiang	46.08	127.4
BY	314	BY137	NER	Bayan, Heilongjiang	46.08	127.4
BY	315	BY145	NER	Bayan, Heilongjiang	46.08	127.4
TB	316	TB149	SR	Tongbai, Henan	32.38	113.42
TB	318	TB138	SR	Tongbai, Henan	32.38	113.42
TB	319	TB144	SR	Tongbai, Henan	32.38	113.42
TB	320	TB136	SR	Tongbai, Henan	32.38	113.42
WQ	321	WQ056	CR	Wuqing, Tianjin	39.38	117.04
WQ	323	WQ112	CR	Wuqing, Tianjin	39.38	117.04
WQ	324	WQ121	CR	Wuqing, Tianjin	39.38	117.04
WQ	325	WQ001	CR	Wuqing, Tianjin	39.38	117.04
WK	326	WK006	NER	Wangkui, Heilongjiang	46.83	126.48
WK	327	WK001	NER	Wangkui, Heilongjiang	46.83	126.48
WK	328	WK004	NER	Wangkui, Heilongjiang	46.83	126.48
WK	329	WK003	NER	Wangkui, Heilongjiang	46.83	126.48
WK	330	WK002	NER	Wangkui, Heilongjiang	46.83	126.48
DQ	331	DQ2512	SR	Deqing, Zhejiang	30.55	119.97
DQ	332	DQ2499	SR	Deqing, Zhejiang	30.55	119.97
DQ	333	DQ2528	SR	Deqing, Zhejiang	30.55	119.97
DQ	334	DQ2526	SR	Deqing, Zhejiang	30.55	119.97
DQ	335	DQ2561	SR	Deqing, Zhejiang	30.55	119.97
AH	336	AH076	SR	Anhua, Hunan	28.38	111.21
AH	337	AH055	SR	Anhua, Hunan	28.38	111.21
AH	338	AH024	SR	Anhua, Hunan	28.38	111.21
AH	339	AH008	SR	Anhua, Hunan	28.38	111.21
JC	341	JC2714	SR	Jucao, Anhui	31.65	117.72
JC	342	JC2700	SR	Jucao, Anhui	31.65	117.72
JC	343	JC2706	SR	Jucao, Anhui	31.65	117.72

JC	344	JC2702	SR	Jucao, Anhui	31.65	117.72
JC	345	JC2722	SR	Jucao, Anhui	31.65	117.72
SFH	346	SFH363	NER	Suifenhe, Heilongjiang	44.41	131.14
SFH	347	SFH362	NER	Suifenhe, Heilongjiang	44.41	131.14
SFH	348	SFH358	NER	Suifenhe, Heilongjiang	44.41	131.14
SFH	349	SFH354	NER	Suifenhe, Heilongjiang	44.41	131.14
SFH	350	SHH356	NER	Suifenhe, Heilongjiang	44.41	131.14
HS	352	HS055	SR	Hengshan, Hunan	27.41	112.62
HS	353	HS003	SR	Hengshan, Hunan	27.41	112.62
WH	356	WH0038	CR	Wuhe, Anhui	33.15	117.88
WH	357	WH054	CR	Wuhe, Anhui	33.15	117.88
WH	358	WH0039	CR	Wuhe, Anhui	33.15	117.88
WH	359	WH060	CR	Wuhe, Anhui	33.15	117.88
WH	360	WH0020	CR	Wuhe, Anhui	33.15	117.88
YC	361	YC2517	CR	Yancheng, Jiangsu	33.35	120.16
YC	362	YC2592	CR	Yancheng, Jiangsu	33.35	120.16
YC	363	YC2591	CR	Yancheng, Jiangsu	33.35	120.16
YC	364	YC2595	CR	Yancheng, Jiangsu	33.35	120.16
YC	365	YC2610	CR	Yancheng, Jiangsu	33.35	120.16
YG	366	YG044	CR	Yanggu, Shandong	36.11	115.79
YG	367	YG036	CR	Yanggu, Shandong	36.11	115.79
YG	368	YG051	CR	Yanggu, Shandong	36.11	115.79
YG	369	YG049	CR	Yanggu, Shandong	36.11	115.79
YG	370	YG052	CR	Yanggu, Shandong	36.11	115.79
LJ	388	LJ075	NER	Longjing, Jilin	42.76	129.42
LJ	389	LJ081	NER	Longjing, Jilin	42.76	129.42
LJ	390	LJ100	NER	Longjing, Jilin	42.76	129.42
LJ	391	LJ084	NER	Longjing, Jilin	42.76	129.42
LJ	392	LJ090	NER	Longjing, Jilin	42.76	129.42
DH	394	DH396	NER	Dunhua, Jilin	43.37	128.23
DH	395	DH391	NER	Dunhua, Jilin	43.37	128.23
DH	396	DH399	NER	Dunhua, Jilin	43.37	128.23

Table S2. Mean genetic distance between locations.

	HS	AH	DQ	JC	TB	WH	YC	YG	WQ	LJ	DH	SFH	HL	YS	BY
**HS**															
**AH**	0.232														
**DQ**	0.257	0.264													
**JC**	0.267	0.279	0.266												
**TB**	0.258	0.269	0.264	0.268											
**WH**	0.299	0.304	0.288	0.309	0.275										
**YC**	0.304	0.308	0.291	0.312	0.276	0.204									
**YG**	0.310	0.316	0.294	0.317	0.277	0.201	0.194								
**WQ**	0.303	0.311	0.287	0.312	0.264	0.194	0.189	0.166							
**LJ**	0.291	0.297	0.287	0.283	0.291	0.319	0.322	0.325	0.316						
**DH**	0.293	0.302	0.287	0.285	0.291	0.322	0.326	0.329	0.319	0.236					
**SFH**	0.275	0.288	0.279	0.269	0.279	0.309	0.312	0.311	0.306	0.228	0.211				
**HL**	0.283	0.298	0.283	0.281	0.288	0.316	0.319	0.319	0.314	0.239	0.224	0.193			
**YS**	0.279	0.291	0.280	0.273	0.281	0.311	0.315	0.314	0.307	0.238	0.217	0.178	0.197		
**BY**	0.286	0.295	0.282	0.275	0.281	0.308	0.307	0.311	0.305	0.267	0.260	0.245	0.258	0.248	
**WK**	0.284	0.296	0.281	0.278	0.285	0.313	0.314	0.317	0.309	0.262	0.262	0.248	0.260	0.250	0.212

Table S3. Selective sweeps between different G.soja groups.

Chromosome	Start	End	Comparison
Gs05	10800001	11300000	CR vs. NER
Gs05	11700001	12200000	CR vs. NER
Gs05	13400001	14100000	CR vs. NER
Gs05	17500001	18500000	CR vs. NER
Gs05	22300001	22900000	CR vs. NER
Gs06	22600001	23500000	CR vs. NER
Gs06	25400001	25900000	CR vs. NER
Gs06	27400001	28100000	CR vs. NER
Gs06	28600001	29100000	CR vs. NER
Gs07	30800001	31300000	CR vs. NER
Gs07	33300001	34200000	CR vs. NER
Gs08	38200001	38700000	CR vs. NER
Gs09	12800001	13500000	CR vs. NER
Gs09	13700001	14500000	CR vs. NER
Gs09	16900001	17500000	CR vs. NER
Gs09	17600001	18600000	CR vs. NER
Gs09	24300001	24800000	CR vs. NER
Gs09	27100001	27600000	CR vs. NER
Gs10	32600001	33300000	CR vs. NER
Gs11	24800001	26100000	CR vs. NER
Gs14	17200001	17700000	CR vs. NER
Gs14	20400001	21000000	CR vs. NER
Gs14	22500001	23000000	CR vs. NER
Gs14	24600001	25300000	CR vs. NER
Gs14	26300001	27100000	CR vs. NER
Gs15	47300001	47900000	CR vs. NER
Gs17	27100001	27600000	CR vs. NER
Gs19	28100001	29300000	CR vs. NER
Gs19	29400001	30100000	CR vs. NER
Gs05	17900001	18500000	CR vs. SR
Gs05	18800001	19300000	CR vs. SR
Gs05	20400001	21200000	CR vs. SR
Gs06	22700001	23200000	CR vs. SR
Gs06	23400001	24000000	CR vs. SR
Gs06	24300001	24900000	CR vs. SR
Gs06	27800001	28400000	CR vs. SR
Gs06	28600001	29100000	CR vs. SR
Gs07	23500001	24400000	CR vs. SR
Gs09	12800001	14500000	CR vs. SR
Gs09	16900001	17400000	CR vs. SR
Gs09	24000001	24600000	CR vs. SR

Gs09	25900001	26600000	CR vs. SR
Gs14	16900001	17700000	CR vs. SR
Gs14	27400001	28000000	CR vs. SR
Gs14	44800001	45300000	CR vs. SR
Gs18	30100001	30600000	CR vs. SR
Gs19	11900001	12400000	CR vs. SR
Gs19	27400001	27900000	CR vs. SR
Gs19	28500001	29400000	CR vs. SR
Gs19	29500001	30300000	CR vs. SR
Gs19	30500001	31000000	CR vs. SR
Gs19	37600001	38300000	CR vs. SR
Gs20	29000001	29500000	CR vs. SR
Gs01	16600001	17300000	NER vs. SR
Gs01	21100001	21900000	NER vs. SR
Gs02	27100001	27600000	NER vs. SR
Gs03	25100001	25600000	NER vs. SR
Gs04	33500001	34000000	NER vs. SR
Gs04	34400001	34900000	NER vs. SR
Gs04	43800001	44300000	NER vs. SR
Gs06	20100001	20600000	NER vs. SR
Gs09	10300001	10900000	NER vs. SR
Gs09	21700001	22200000	NER vs. SR
Gs10	17500001	18000000	NER vs. SR
Gs10	18200001	19100000	NER vs. SR
Gs10	19700001	20400000	NER vs. SR
Gs10	20700001	24000000	NER vs. SR
Gs10	31200001	31700000	NER vs. SR
Gs11	7800001	8300000	NER vs. SR
Gs11	25800001	26400000	NER vs. SR
Gs12	10000001	10800000	NER vs. SR
Gs13	21700001	22200000	NER vs. SR
Gs13	22200001	23100000	NER vs. SR
Gs14	10700001	11200000	NER vs. SR
Gs14	20400001	21000000	NER vs. SR
Gs14	22000001	23000000	NER vs. SR
Gs14	24600001	25100000	NER vs. SR
Gs14	25400001	26000000	NER vs. SR
Gs14	26200001	27100000	NER vs. SR
Gs14	28600001	29100000	NER vs. SR
Gs14	31300001	31800000	NER vs. SR
Gs14	31900001	32600000	NER vs. SR
Gs14	39800001	40300000	NER vs. SR
Gs15	23600001	24300000	NER vs. SR

Gs15	39500001	40400000	NER vs. SR
Gs15	47100001	47900000	NER vs. SR
Gs16	22000001	22500000	NER vs. SR
Gs17	2700001	3300000	NER vs. SR
Gs17	19300001	20300000	NER vs. SR
Gs17	21900001	22500000	NER vs. SR
Gs18	13800001	14500000	NER vs. SR
Gs18	14700001	15300000	NER vs. SR
Gs18	17000001	17900000	NER vs. SR
Gs18	22800001	23400000	NER vs. SR
Gs18	34100001	34600000	NER vs. SR
Gs18	34700001	35200000	NER vs. SR
Gs18	37400001	38200000	NER vs. SR
Gs18	38500001	39100000	NER vs. SR
Gs19	17400001	18000000	NER vs. SR
Gs19	19200001	19800000	NER vs. SR
Gs20	19600001	20100000	NER vs. SR
